# Problematization of perspectives on health promotion and empowerment in mental health nursing—Within the research network “MeHNuRse” and the Horatio conference, 2012

**DOI:** 10.3402/qhw.v9.22945

**Published:** 2014-04-08

**Authors:** Patrik D. Jönsson, Håkan Nunstedt, Inger J. Berglund, Britt H. Ahlström, Birgitta Hedelin, Ingela Skärsäter, Henrika Jormfeldt

**Affiliations:** 1 Department of Research, Development and Education (FoUU), Region of Halland, Halmstad, Sweden; 2 School of Social and Health Sciences, Halmstad University, Halmstad, Sweden; 3 Department of Nursing, Health and Culture, University West, Trollhättan, Sweden; 4 Skaraborg Hospital, Skövde, Sweden; 5 Department of Nursing, Gjøvik University College, Gjøvik, Norway

**Keywords:** Emancipation, empowerment, health promotion, mental health nursing, self-efficacy, self-management

## Abstract

Mental illness is increasing worldwide, while society's response seems to be a trend toward narrower and more specialized mental health care. This development is creating great demands on mental health nurses to include a health promotion perspective in care and support of persons with mental illness. A health promotion perspective emphasizes cooperation and communication with people who suffer from long-term mental illness, focusing on their independence and health. From a health perspective, every human being is an actor in his/her own life, with an inherent ability to make his/her own choices. However, persons who suffer from long-term mental illness are at risk of losing power and control over areas of their lives and their health. Mental health nurses are in a position to support these individuals in promoting health and in maintaining or regaining control over their lives. The emphasis of this paper is to problematize mental health nurses’ responsibility to provide health-promoting nursing care in relation to empowerment by means of emancipation, self-efficacy, and self-management. We argue that mental health nurses can work from a health-promoting perspective by using these concepts and that this challenges some of the traditional ideas of health promotion in mental health nursing. The theoretical background discussions in this paper have their origin in the research network “Mental Health Nursing Research in Scandinavia” (MeHNuRse) and from the professional discussions developed during a 2012 workshop that included mental health nurses and researchers at the European Horatio Festival in Stockholm.

From a holistic health perspective, every human being is an actor in his/her own life, with an inherent right and ability to make his/her own choices. However, individuals with a long-term mental illness are at risk of losing power and control over areas of their lives, depending on the impact that the mental illness has had on the person's functioning and perception of self (Hansson, Jormfeldt, Svedberg, & Svensson, 2011). To experience health, it is crucial to have equality, a meaningful life, and freedom of choice. Patients in mental health services have previously described that a mental illness limits their opportunities for personal development and health. To achieve a feeling of freedom in spite of or independent of the disease, the courage is needed to take chances and steps toward change (Svedberg, Jormfeldt, Fridlund, & Arvidsson, 2004). Furthermore, in many cases patients, with different mental health conditions believe that they have only weak influence over their treatment and life situation, and they perceive that the information they are given regarding treatment alternatives is incomplete (Swedish Council on Health Technology Assessment [SBU], 2011).

These circumstances put great demands on nurses to work from a perspective that emphasizes cooperation and communication with persons suffering from long-term mental illness and with a focus on the persons’ experience of independence and health. From this perspective, the development of health-promoting nursing care should emphasize strengthening patients’ ability to make their own choices and to influence and control their treatment and life situation, thereby allowing for more effective self-care. This is also what the World Health Organization (1986) has defined as the cornerstone of empowerment. In health care, there is a need to develop and implement methods that respect the patient's voice because as systematic knowledge of the effects of these methods is still scarce (SBU, 2011).

In recent years, health-promoting educational interventions have been scientifically studied and have been found to support equality, participation in decision making, increased self-esteem, and self-management ability. The educational and didactic role in health-promoting nursing is central, but the models for the role in relation to these concepts needs to be further developed and problematized (Anderson & Funnell, 2005; Jönsson, 2010; Jormfeldt, Brunt, Rask, Bengtsson, & Svedberg, 2012; Jormfeldt, Rask, Brunt, Bengtsson, & Svedberg, 2013).

Two traditions of health education have been described in the literature—a medical model and an education model. In the medical model, the purpose of health education is to develop effective interventions that will prevent people from adopting unhealthy behaviors. According to the medical model, health educators are expected to replicate the methods identified by researchers to effect targeted changes in health behavior. In the educational model, the purpose of research and practice is to clarify basic social values and to strengthen peoples’ faculty for making value judgments (Buchanan, 2006). The process of increased growth and subjectively experienced health, which has been labeled transition, is encouraged through communication of thoughts and needs (Skärsäter & Willman, 2006). Practitioners adopting a philosophy of education use research results as a motivation for dialogue. The increased personal growth is connected to healthier lifestyle behaviors in accordance with individual goals. Dimensions of health have to be promoted at the individual level (Mezzich, 2005) and include factors such as acceptance, faith, hope, meaningfulness, and meaningful relationships (Edward, Welch, & Chater, 2009).

Through their caring assignments, mental health nurses have a responsibility to enable the empowerment of persons with long-term mental illness. One primary objective of the educational and didactic function of nurses is to support these individuals in recognizing, understanding, and using their own resources. Also inherent in this empowerment process is support of the individuals’ emancipation, self-efficacy, and self-management. Mental health nurses need a common language and a mutual understanding of essential concepts in order to develop culturally sensitive approaches that secure individuals’ rights regarding health promotion in mental health care.

The emphasis of this paper is to problematize mental health nurses’ responsibility to provide health-promoting empowerment by means of emancipation, self-efficacy, and self-management. Furthermore, we pay attention to mental health nurses’ and researchers’ views of these common mental health issues from an international perspective. This discussion was raised during a workshop on health-promoting nursing care and the educational function of mental health nurses held in 2012 at Stockholm's Horatio Festival. Mental health nurses and researchers from more than 20 countries participated in the conference.

## Essential concepts of health-promoting empowerment in mental health nursing

Mental health nurses’ knowledge about their responsibility to provide health-promoting care can develop through a common understanding of empowerment by means of emancipation, self-efficacy, and self-management. In this section, we describe how these concepts support empowerment from the mental health nursing perspective.

### Empowerment

Empowerment can be described as the overall process of increased self-esteem, which is essential for individuals to gain the energy needed to fully develop their personal health capacity (Anderson & Funnell, 2005; Johnson, 2011; Jones & Meleis, 1993). Persons with a mental illness participating in person-centered education have described positive health experiences in relation to essential aspects of empowerment (Jormfeldt et al., 2012). Empowerment encompasses these individuals’ responsibilities regarding their own health. Furthermore, it encompasses broader organizational and societal responsibilities by enabling people to assume responsibility for their own health. From a user perspective, a relationship needs to be built on respect, involvement, and participation in decision making, which are the fundamentals in processes of empowerment (Nygårdh, Malm, Wikby, & Ahström, 2012; Nygårdh, Wikby, Malm, & Ahlström, 2011). According to mental health staff, empowerment processes in mental health care and services are dependent on financial priorities and on the sharing of responsibilities at an organizational level (Jormfeldt et al., 2013).

### Emancipation

Emancipation literally means to “give away ownership…set free, especially from legal, social, or political restrictions…setting free, delivering from intellectual, moral, or spiritual fetters” (Oxford English dictionary online, 2013). More broadly, it means to relinquish one's authority over someone else. The object of emancipation—that is, the person to be emancipated—becomes independent, or as independent as possible, and as free as possible with respect to their autonomy and self-chosen activities as a result of the act of emancipation. To achieve emancipation requires an intervention by someone who is not subjected to the power that needs to be overcome (Bingham & Biesta, 2010). Emancipation is gained by learning the processes of reflection and self-reflection; new knowledge and understanding can free an individual from previously hampering conceptions (Gergen, 1999).

Patient emancipation involves setting the patient free from the control of those more powerful, such as nurses, doctors, and other authorities (Brown, 1993; Williamson, 2008). Thus it is important that patient emancipation movements are characterized by questions of arrangements between groups and how to balance the power between them. For a person with mental illness, greater knowledge and understanding of the situation can support individual emancipation and increased self-management (Nunstedt, Nilsson, Skärsäter, & Kylén, 2012). The processes of emancipation enable the person to become aware of his/her own potential by finding inherent knowledge, values, motivations, and goals, and by linking these factors to actions.

### Self-efficacy

Self-efficacy concerns a person's beliefs about his/her capability to create designated levels of performance and to exercise influence over different life events (Bandura, 1997, 2002). Self-efficacy involves beliefs that determine how the individual feels, thinks, motivates him/herself, and behaves. Those who have high confidence in their capabilities approach demanding tasks as challenges to be mastered, rather than as threats to be avoided (Zimmerman, 2000). Persons with self-efficacy set challenging goals for themselves, maintain strong commitment to these goals, and intensify and sustain their efforts in the face of failure. They quickly recover their sense of efficacy after failures or setbacks, and they attribute failure to insufficient effort or to deficient knowledge and skills that are acquirable. Persons with self-efficacy tackle threatening situations with the understanding that they can master them and have control over them. Such an efficacious outlook produces personal accomplishments in the area of mental illness because it has been shown to reduce stress and lower vulnerability to depression. In relation to mental illness, it is common for individuals to experience hopelessness and a perceived inability to influence life (Hansson et al., 2011; Jönsson, Wijk, Skärsäter, & Danielson, 2008); thus knowledge of the impact and significance of self-efficacy may raise new possibilities in mental health nursing from a health promotion perspective.

### Self-management

Self-management is one of the key developments that allows persons with long-term mental illness to be able to manage on their own and to be independent of others within the context of care and treatment that traditionally is entirely controlled by clinicians (Crepaz-Keay, 2010). The concept of self-management is about the person's ability to manage the symptoms, treatment, physical and psychosocial consequences, and lifestyle changes inherent in living with a long-term condition. Efficacious self-management encompasses the ability to monitor one's condition and to affect the cognitive, behavioral, and emotional responses necessary to maintain a satisfactory quality of life. Learning about responses to illness is mainly done through experiences of trial and error in daily life (Kralik, Koch, Price, & Howard, 2004). Self-responsibility and self-control are meaningful values in the activities and decisions of the daily life of a person with a long-term illness (Delmar et al., 2005). Furthermore, having dignity and being respected as an individual are closely connected to being able to self-manage. Through learning from daily life experiences, a person′s ability to make changes to their daily life and their self-identity is reconstructed by exploring personal limitations or boundaries. Self-management of long-term illness contains both structure and process, and it is the process of self-management that is central to the experience of moving from a stressful and uncertain life toward normality and the manageability of life with an illness (Kralik et al., 2004). Self-management in mental health care is about transferring the focus from treating a condition or illness to enabling people to live with that condition or illness over the long term. This transferred focus enables people to rebuild their lives within the context of living with mental illness and is a structured approach to transferring control back to individuals (Crepaz-Keay, 2010).

## European mental health nurses’ views of empowerment related to actual concepts in health-promotion

A two-hour workshop composed of an initial lecture and then small group discussions in the auditorium was carried out at the 2012 Horatio Festival in Stockholm. Sixty-one nurses and researchers attending the workshop during which the following question was raised and discussed: Why should mental health nurses pay attention to the interpretations of empowerment through the concepts of emancipation, self-efficacy, and self-management within the context of mental health/illness? During group discussions in the auditorium, understanding of the described concepts was related to health and health-promoting nursing and the discussions were noted and summarized in three main themes: mental health nurses’ competence to judge patients’ needs, mental health nurses’ tools to support empowerment, and organizational power versus patient empowerment.

### Mental health nurses’ competence to judge patients’ needs

The discussions encompassed mental health nurses’ responsibility to provide patients with possibilities for participating in social activities and sports exercise. Group members maintained that mental health nurses have a responsibility to actively participate in supporting patients in mental health care and in services using the range of health-promoting opportunities and activities that are offered in society. Experiences shared by the group members indicated that these types of resources were not frequently used among patients in mental health care and services. Thus it is important that nurses are active in this regard and meet their patients on the patients’ terms. The groups discussed how to handle different degrees or levels regarding the individual service users’ ability to move toward self-empowerment. One issue was how mental health nurses can identify where the patient is positioned regarding the degree of emancipation. Sometimes mental health nurses need to take control or supervise the patient. Group members raised the question, “How could the degree of emancipation and empowerment of the individual service users be assessed and in relation to what should it be assessed—the organization, expected goals in society, or individual needs?” The groups concluded that an organization's management needs to be able to trust that the nurse in mental health care has the competence to judge when to offer support and when to give space for the service user to take charge of his/her life. One major problem described during the group discussions was the health care organization representatives’ and staffs’ tendencies to neglect the importance of patients’ personal relationships in their recovery processes. To be able to choose friends and neighbors with regard to individual preferences was seen as an important part of self-management.

### Mental health nurses’ tools to support empowerment

The discussions included the question of what a mental health nurse can do to support the service user in his/her emancipation. During the discussions, participants maintained that the most frequently used tool to enable and facilitate emancipation and empowerment among service users was communication—through dialogue and in a trusting relationship. The importance of meeting the patient on an equal level and being able to adjust actions to individual needs was discussed. One opportunity for mental health nurses to meet patients with special needs was to socialize with the patients in different places if they did not want to meet the nurses at the nurse's office. To meet a patient in other places than in mental health services is a possibility for the mental health nurse to moderate the imbalance of power in the relationship between the nurse and the patient. Thus helping with practical matters such as walking the patient's dog together with the patient, instead of simply talking about and showing interest in the patient's mental health problems, could be a way to build a trusting relationship and alliance. This kind of activity in building relationships was described as more challenging in in-patient care. Discussion participants stated it was essential to create room for the patients’ decisions, even small ones. To build empowerment by supporting self-efficacy, self-management, and participation in decision making was described as most important, even if the decisions objectively judged were not healthy at first. Group members concluded that the use of interventions that employ the principles of emancipation and empowerment needs to be further developed. During the discussions, participants maintained that staff does not always let patients make their own decisions or set individual goals regarding their care.

### Organizational power versus patient empowerment

Finally, the discussions covered the health care organizational dimension of patient empowerment by highlighting the issues of power imbalance that might exist in the relationship between the service user and the health care organization. The discussion participants embraced the importance of role models and parallel processes in health care organizations to promote human development. Group members stated that nurses need to be empowered in their organization to in turn be able to empower patients in the nursing relationship. Nurses can, for example, be empowered in dialogue with colleagues. Also, organizations need competent leaders who are open to listening to staff and service users. That is necessary if the health care organizations are to develop and become able to sufficiently adjust to the patients’ emancipation, self-efficacy, and self-management in terms of opening hours, continuity in nurse-patient relationships, and so on. The group members finally emphasized the need to define and share the same conceptual basis and core values within an organization and reported that therapeutic optimism is needed. Organizations have to pay attention to how the professionals’ routines and treatments affect patients’ lives because the staff has both a direct and an indirect influence on the service users’ daily lives. However, the participants said that this form of assessment and development is rarely seen in praxis.

## Summary and outlook

The group discussions among the mental health nurses and researchers in the workshop at Stockholm's 2012 Horatio Festival revealed obvious interest in a common international language for health-promoting mental health nursing. In the group discussions, the participants addressed three themes: mental health nurses’ competence to judge patients’ needs, mental health nurses’ tools to support empowerment, and organizational power versus patient empowerment. These themes were perceived as closely related to health promotion, and the concepts of emancipation, self-efficacy, and self-management were perceived to highlight what is characteristic for empowerment in mental health nursing and the role of mental health nurses in the care of individuals with long-term mental illness. The discussions ended with questions about how the significance of empowerment in mental health nursing could be more clearly expressed by using the concepts of emancipation, self-efficacy, and self-management. The discussions also supported the assumed suggestion that emancipation, self-efficacy, and self-management are useful concepts for mental health nurses who every day work to support patient empowerment. The participating mental health nurses and researchers expressed a need to further discuss the relation between the concepts and empowerment and to find better methods and outcomes in mental health care from a user perspective. Their hope was that the discussions taking place at the workshop might be a starting point for developing more health-promoting mental health care. This paper can serve as a foundation for further discussions and studies on health—promotion and empowerment and the importance of emancipation, self-efficacy, and self-management within mental health nursing. With a greater understanding among mental health nurses and application of empowerment through these concepts, more health-promoting care can be provided to persons with long-term mental illness.
